# Foot Morphology and Substrate Adhesion in the Madagascan Hissing Cockroach, *Gromphadorhina portentosa*

**DOI:** 10.1673/031.010.4001

**Published:** 2010-05-08

**Authors:** Adam van Casteren, Jonathan R. Codd

**Affiliations:** Faculty of Life Sciences, University of Manchester, Manchester, UK

**Keywords:** attachment pads, Instron® universal testing machine, peak force, pretarsal claws, tarsal structure

## Abstract

Insects are successful terrestrial organisms able to locomote over a wide range of obstacles and substrates. This study investigated how foot morphology (tarsal structure) correlates with substrate adhesion and ecological niche in the Madagascan hissing cockroach, *Gromphadorhina portentosa* Schaum (Blattaria: Blaberidae). Using light and scanning electron microscopy, the morphology of the different structures of the tarsus of *G*. *portentosa* was analysed. Using an Instron® universal testing machine, a series of peak force experiments were then conducted to record the force required to lift the cockroaches off different substrates. *G. portentosa* was pulled off 10 different substrates, which consisted of smooth Perspex; Perspex scored at 1cm intervals; Perspex hatched at 1 cm, 0.5 cm, and 1 mm intervals; Perspex abraded with fine grade sandpaper; Perspex abraded with coarse grade sandpaper; wood; glass; and Teflon. A clear relationship was seen where an increase in scoring on the Perspex caused a decrease in adhesive ability of *G. portentosa*. This may be due to there being adequate contact area for the attachment of the pads and to allow the claws to engage. The results obtained suggest that to achieve the greatest adhesion to substrates, *G. portentosa* uses a combined effect of both adhesive pads and pretarsal claws. Adhesion to a wide range of substrates appears to be an adaptation to life as a wingless forest floor dweller.

## Introduction

Insects use hooks or spines to adhere to rough substrates; however, when these structures cannot engage on smoother surfaces, attachment pads are used (Arnold 1974; Walker 1992; Fraizer 1999; Goodwyn et al. 2006). These attachment pads must adhere successfully to the substrate but also must have the capacity to be readily and easily detached (Gorb and Scherge 2000; Gorb et al. 2002). Attachment pads vary greatly in morphology but commonly are composed of a hairy surface or a smooth flexible pad. Both types of pad are adapted to maximize contact with the diverse array of substrate with which insects potentially come into contact. This then allows the insect to rapidly locomote while still gaining a firm adhesion to the substrate (Walker 1992; Gorb et al. 2000; Votsch et al. 2002; Drechsler and Federle 2006; Goodwyn et al. 2006).

The hairy pad system used by flies, beetles, and earwigs consists of many deformable seta that allow a maximal contact with the substrate giving the adhesive quality (Votsch 2002; Niederegger et al. 2002; Langer et al. 2004). The smooth pad system that is used by cockroaches, grasshoppers and bugs is a soft deformable pad that mimics the contours of the substrate allowing maximal contact. (Jiao et al. 2000; Beutel and Gorb 2001; Gorb et al. 2002; Federle et al. 2002; Drechsler and Federle 2006). The deformable pad is made up of mainly endocutical, with a fibrous structure. Under pressure these fibres can flatten and move close together allowing the pad to mimic the substrate, while as pressure is released, the fibers return to their original position (Jiao et al. 2000; Beutel and Gorb 2001; Goodwyn et al. 2006). The construction of the pad enables it to provide stability as well as being highly flexible, meaning the pad will deform to match the substrate, therefore allowing the insect to adhere to a range of substrates found in mobile terrestrial life (Roth and Willis 1952; Gorb et al. 2000; Jiao et al. 2000; Beutel and Gorb 2001; Gorb and Beutel 2001; Gorb et al. 2002).

In both forms, pad adhesion is facilitated by small amounts of fluid that is secreted into the area of contact. The fluid does not solely account for the adhesive ability of insects, but it is necessary for adhesion to occur (Jiao et al. 2000; Votsch et al. 2002). The fluid secreted by the pads is a two phase hydrophobic and hydrophilic liquid (Jiao et al. 2000; Votsch et al. 2002; Federle et al. 2002; Drechsler and Federle 2006). it is an emulsion made up of a water soluble fraction and lipid-like nano-droplets (Votsch et al. 2002). The water soluble part of the secretion contains carbohydrates, consisting mainly of glucose, but xylose and mannose are also found in lower concentrations. It is also presumed that amino acids and proteins are present in the secretion, which could act to increase viscosity of the fluid; but there is no definitive evidence to support this, and it is unclear whether these elements are deliberately added to the liquid or if they occur simply as contamination of the compounds (Votsch et al. 2002). The lipid-like nano-droplets of the secretion most likely are made up of fatty acids (Votsch et al. 2002). The mechanism by which this fluid secretion aids adhesion is not yet fully understood, but there are a variety of explanations within the literature. The simplest explanation was that the fluid itself acted as a kind of sticky glue, but this has been largely discredited (Jiao et al. 2000; Votsch et al. 2002). There is another theory of “wet adhesion” whereby the viscosity of the fluid aids adhesion of the pad to the substrate (Federle et al. 2002; Votsch et al. 2002; Drechsler and Federle 2006). A further theory is that the fluid helps in providing greater attachment to rough substrates. The fluid fills the cavities in a rough substrate allowing the smooth deformable pad to gain maximal contact with the surface where a dry pad would only make limited contact (Drechsler and Federle 2006). These types of secretions are described in many cockroach species (Roth and Willis 1952; Arnold 1974), and therefore it can be assumed to facilitate the adhesive abilities of *Gromphadorhina portentosa* Schaum (Blattaria: Blaberidae).

*G*. *portentosa,* like all cockroaches, possesses smooth flexible pads found at the distal end of the leg on the tarsus. There are five pads altogether; the first four segments of the tarsus contain pads called the euplantula, while the most flexible fifth segment found at the distal end of the tarsus and is called the pretarsus, which is made up of a broadly triangular adhesive pad called the arolium and two pretarsal claws (Roth and Willis 1952; Dailey and Graves 1976). The euplantula and arolium have been shown to have different uses during terrestrial locomotion. The euplantula are used when the legs are pushing, in walking and climbing, and have been demonstrated not to act as adhesive organs but rather in fact as friction pads used to power locomotion (Clemente et al. 2008). The pretarsal claws allow cockroaches to grip and move over rough surfaces, and the arolium is the adhesive organ of the cockroach foot engaged when the legs are pulling and used in scaling and adhering to smooth surfaces (Arnold 1974; Dailey and Graves 1976; Clemente et al. 2008).

There are relatively few studies on the adhesive ability of live insects, and these tend to focus on the hairy pad system of adhesion. Here, light and electron microscopy are used in combination with biomechanical testing to test the hypothesis that the adhesive ability of *G*. *portentosa* is related to substrate morphology.

## Methods

### Cockroach husbandry

Experiments were conducted upon five adult, female cockroaches of the species *G. portentosa.* Females were used as aggression by males in the colony can lead to tarsal damage, and males commonly were missing one or more tarsa. *G*. *portentosa* were kept in a tank (35 cm × 20 cm × 22 cm) at room temperature (≈ 18–22° C). The tank had approximately 2 cm of orchid bark substrate, and cardboard rolls and tree stumps were provided for housing. *G*. *portentosa* were fed a combination of vegetable matter with dried dog food for protein. Food and water were provided *ad libitum.*

### Microscopy

Light microscope pictures were taken using a Leica MZ9s (www.leica-microsystems.com) stereo microscope. The images were then processed using Leica Application Suite, which allowed morphological measurements to be taken. The cockroach leg was then desiccated by placing the samples on gauze above silica gel for at least a week. Prior to slide preparation for scanning electron microscopy (SEM), the sample was washed in ethanol to remove any dirt or dust, coated with a fine layer of gold, and scanned. This process was carried out for two legs from one cockroach, a front and a rear leg, as the middle and rear legs are structurally identical (Watson and Ritzmann 1998).

### Mechanical Testing

**Figure 1.**
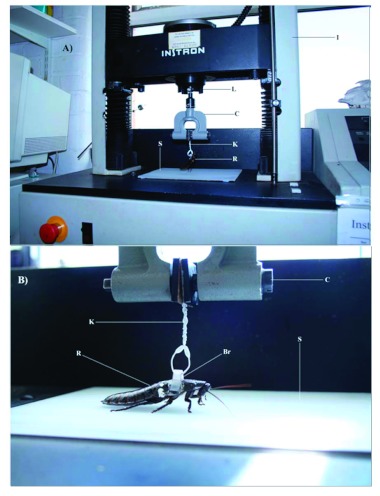
Experimental setup for peak force experiments. A) Instrom universal testing machine, B) Close-up of attachment of *Gromphadorhina portentosa* to the load cell. Labels: C = Clamp, S = Sample substrate, Br = Bracket, K = Kitchen twist tie, R = *G*. *portentosa*, L = Load cell, I = Instron® machine. Pictures taken by AvC. High quality figures are available online.

*G*. *portentosa* were briefly anaesthetized by exposure to CO2 for three minutes. Then a specially constructed plastic bracket (5 × 28mm) was fixed using cyanoacrylate glue to the second thoracic section over the middle pair of legs (Figure 1B). The site of attachment was cleaned using ethanol to remove the waxy cuticle. The bracket was positioned so as not to interfere with the insects' movement or ability to adhere to the substrate as there was no interference with the legs or hindrance of any body flexion. Once the bracket had been attached, the cockroach was left for at least 5 h for a period of recovery prior to experimentation (Storke 1980).

The force needed to remove the cockroach from the substrate was calculated using an Instron®, (www.instron.com) universal testing machine. This equipment is commonly used to measure tensile or compression forces via a load cell and generates highly accurate and repeatable measures (Vincent 1992). The cockroaches were pulled at a constant speed and the peak force generated during the pull was recorded. A 14 cm piece of plastic twist tie was threaded through the bracket and twisted together to create a loop around the bracket and then inserted into a clamp that hung from the 100N load cell (Figure 1A). Each cockroach (*n* = 5) was pulled twenty times from each of the ten different substrates, at a constant speed of 50 mm/second. *G. portentosa* was allowed a settlement period of 5 s between pulls, allowing sufficient time for pad attachment to the substrate (Drechsler and Federle 2006). When each pull commenced, it continued until all legs of the cockroach were removed from the substrate. From each pull, the peak force was calculated by an interfacing computer and recorded. *G. portentosa* was pulled from 10 different substrates: smooth Perspex, 1 cm vertically scored Perspex, hatched Perspex (1, 0.5, 0.01 cm), roughened Perspex (fine & coarse), glass, wood, and Teflon. The experimental adhesion testing was filmed using a digital camera (Nikon Coolpix S10, www.nikon.com), see Video.

**Figure:**
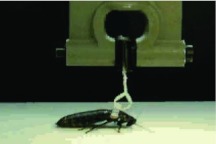
Video of experimental protocol during adhesion testing of *Gromphadorhina portentosa* using the Instron® universal testing machine. Filename: vancasteren_codd_experimental_testing The video file is available online.

### Substrate Construction

To determine the effect of surface smoothness on adhesive ability, Perspex and glass substrates were used. These were both purchased from commercial sources. To assess the implications of increasing roughness on adhesion, the Perspex was subjected to increasing degrees of scoring. The scoring of the Perspex was conducted by hand using a razor blade 0.22 × 10-3m wide and a metal ruler. Using moderate force, the razor blade was drawn across the Perspex in one smooth action, generating the score. To enable results to be compared to substrates that were more representative of those the cockroaches may encounter in the natural habitat, testing was also conducted on wood substrate. The wood was a deciduous hardwood log (Oak, *Quercus robur*) obtained from nearby woodland. The bark was removed, and the wood autoclaved to remove any foreign influences that could affect adhesion. The shape of the wood was not altered. To assess the influence of surface polarization, a Teflon-covered substrate was constructed from a non-stick baking tray.

### Statistics

A one-way ANOVA was performed to determine if there was any difference between cockroaches on each substrate. The statistical test showed that there was no significant difference between each substrate. Data were analyzed using SPSS for windows (version 13.0). There was no significant difference between cockroaches; therefore, data were pooled for each substrate. As the data were not normally distributed, a non-parametric Kruskall-Wallis test was conducted. A Nemenyi post-hoc test was then run to determine which substrates were significantly different from each other.

## Results

### Microscopy

**Figure 2.**
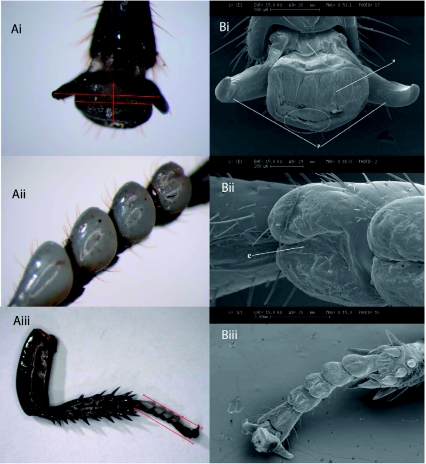
A) Light (LM) and B) scanning electron (SEM) images of i) Pre-tarsal organ (magnification 40× LM, S3.1× SEM) ii) Euplantula (mag. 30× LM, 88.0× SEM) iii) Entire foot of *Gromphadohina portentosa* (mag. 6.3× LM, 15.9× SEM). Red lines indicate measurements taken, white circle demonstrates area where “finger-like projections” were found. Labels; a = Arolium, p = Pretarsal claws, and e = Euplantula. High quality figures are available online.

**Figure 3.**
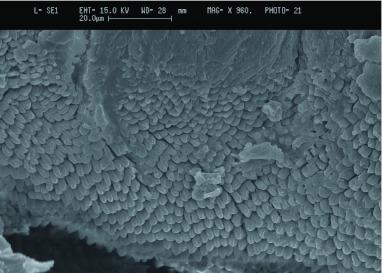
Scanning electron microscope image of the arolium of *Gromphadorhina portentosa* demonstrating the finger-like projections near the edge of the arolium. High quality figures area available online.

The light microscope pictures allowed closer examination of the different structures of the *G*. *portentosa* tarsus. It was possible, for example, to observe the euplantula pads and pretarsus in great detail. Using the microscope allowed measurements of the adhesive organs to be made so it could be assessed whether size had an influence upon the adhesive ability of *G*. *portentosa.* SEM pictures of each of the pretarsal structures clearly showed the smooth arolium between the pretarsal claws (see Figure 2). Under closer examination at higher magnification of the arolium of *G*. *portentosa,* it was possible to see finger-like projections near the edge of the arolium (circled area in Figure 2), these projections are clearly seen in Figure 3. The “finger-like” projections were also seen on the tarsus of the front leg although it appeared some damage may have occurred to the arolium during the desiccation process and were thus not as clear as shown on the rear leg. SEM pictures of the euplantula of *G*. *portentosa* also showed the smooth structure of the pads as well as some interesting structural folds upon the pads (Figure 2).

### Substrate adhesion

**Figure 4.**
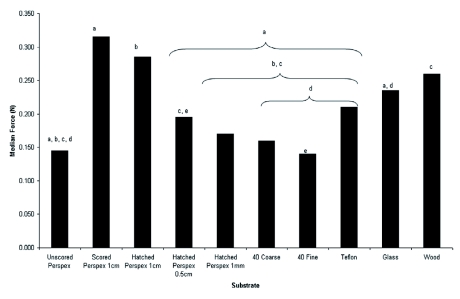
Median peak force recorded for each substrate tested. Significant differences are illustrated by letter pairs. High quality figures area available online.

The results from the Kruskal-Wallis test indicated that there was a significantdifference between the means (F = 16.981, p < 0.001). The Nemenyi post-hoc test revealed that a significantly greater peak force was recorded when *G. portentosa* was pulled from Perspex (see video in supplementary material) which had been scored along the Y axis at 1cm intervals (p < 0.05) than when *G*. *portentosa* were pulled from smooth Perspex, Perspex hatched at 0.5 cm intervals, Perspex hatched at 1 mm intervals, Perspex rubbed 40 times with fine grade sandpaper, Perspex rubbed 40 times with coarse grade sandpaper, glass, or Teflon. Perspex that had been hatched at 1 cm intervals had significantly higher peak force and Teflon. Wood showed similar significance to Perspex hatched at 1 cm; recordings (p < 0.05) than smooth Perspex, Perspex that had been hatched at 1 mm intervals, Perspex rubbed 40 times with fine grade sand paper, Perspex that had been rubbed 40 times with coarse grade sandpaper, however, wood was significantly different from Perspex hatched at 0.5 cm (p <0.05). Glass was significantly different (p < 0.05) from Perspex rubbed 40 times with coarse sand paper, Perspex rubbed 40 times with fine sand paper, and Teflon. The median peak force with Perspex hatched at 0.5 cm was significantly greater than with Perspex rubbed 40 times with fine sand paper. There were no significant differences between smooth Perspex, Perspex hatched at 1 mm intervals, Perspex rubbed 40 times with fine grade sandpaper, Perspex rubbed 40 times with coarse grade sandpaper, or Teflon. As the degree of scoring on the Perspex increased, there was a concomitant decrease in the mean peak force recorded for that substrate (Figure 4).

## Discussion

Previous work on the relationship between surface roughness and adhesive ability has shown that with an increase in surface roughness can lead to a decrease in adhesive ability (Huber et al. 2007) and this would explain the pattern demonstrated in figure 4. As the scoring increased the surface roughness of the Perspex the median peak force recorded showed a significant decline. The significantly higher peak force measured on the scored Perspex (1cm) could be explained due to the combined effect of adhesive pads and pretarsal claws. The morphological analysis indicated that when attempting to adhere to the scored Perspex the cockroaches would have been able to use the pretarsal claws to hook into the scores whilst also using the adhesive pads of the tarsus (euplantula and arolium) to make maximal adhesive contact with the substrate. As the distance between the scores decreased there was less chance that the adhesive pads of the tarsus have sufficient space for effective adhesive contact with the substrate. Surface roughness can influence adhesion, the increased scoring would decrease the maximal contact with the substrate that is needed for effective adhesion of the pads to occur (Jiao et al. 2000, Beutel and Gorb 2001). An increase in the degree of scoring would have also increased the number of potential attachment sites for the claws and it is only if the claws fail to engage that they are pushed out of the way by smooth surfaces which then allows arolium to come into contact with the substrate (Arnold 1974, Fraizer 1999, Goodwyn et al. 2006). The combined effect of available attachment sites for the claws and the available space for pad attachment may have resulted in a higher peak force recordings seen for scored Perspex at 1cm intervals (Roth and Willis 1952, Beutle and Gorb 2001). However to confirm this hypothesis there would have to be clear demonstration of the role of each of the tarsal structures on the differing substrates.

There was no significant difference in median peak force between the smooth Perspex and the Perspex surfaces abraded with sandpaper. In both cases, abrasion of the Perspex surface by sandpaper likely did not provide the roughness required for the tarsal claws to engage and enable greater adhesion. Even after abrasion, the Perspex may have still been smooth enough to push the claws out of the way, and, if this is the case, adhesion would have relied solely upon the arolium, much the same effect as would be found on smooth Perspex. However, this hypothesis remains to be tested through further work from claw clipping experiments such as those seen in Voight et al. (2008). The smooth surface of Teflon did not yield a significantly different mean peak force from that of smooth Perspex or any of the Perspex abraded with sandpaper. This may mean that only the arolium was involved in adhesion. Furthermore, there were no significant differences among the smooth surfaces of the Perspex, abraded Perspex, Teflon, and the regularly scored surfaces of hatched Perspex at 1 mm. On all of these surfaces the possibility exists that only one of the pretarsal adhesive strategies could be used, and this may have been responsible for the significantly lower median peak forces recorded.

When *G*. *portentosa* were pulled off the wood substrate, there was no significantly different mean peak force from scored Perspex (1 cm). *G*. *portentosa* was tested on wood as it was believed it would represent a more relevant substrate because *G*. *portentosa* is a forest floor dwelling cockroach and therefore often encounters wood in its natural habitat (Darmo and Ludwig 1995). The wood sample used in these experiments had the outer layer of bark removed and was therefore relatively smooth. It was hard to quantify the surface of wood in the same manner as for the Perspex, but it is likely that it had similar properties due to the fact that both pads and claws could have been involved in adhesion. Scored and hatched Perspex (1 cm) provided well-spaced ridges for claws to engage and ample smooth surface for the use of attachment pads. Wood did not have the regulated scores of the Perspex substrates, but wood is softer than Perspex likely allowing the claws to imbed into the wood while the attachment pads still had had ample room to facilitate the significantly greater mean peak forces generated on the wood substrate.

On Teflon, Perspex abraded with fine sand paper, and Perspex abraded with coarse sandpaper, a running action was observed as the tests began, which is most vigorously seen on the Teflon surface. However, this effect is not seen as much when *G*. *portentosa* was located on glass and smooth Perspex, probably due, in part, to van der Waals forces. These inter-molecular forces are strongly dependant upon the distance between and polarizability of the two surfaces (Autumn 2006). As Teflon is a non-polarized material, van der Waals forces should not have occurred, making it harder to make adhesive contact between the attachment pads and the surface; the abrasions found on the Perspex surfaces that had been rubbed by sandpaper probably reduced the microscale contact formed by the attachment pads with the substrate, and this could have reduced the van der Waals forces between the cockroach and the substrate. The lack of van de Waals may have induced the frantic running seen at the start of these tests on smooth surfaces and could have explained, in part, the significantly higher mean peak force observed in glass as opposed to in other surfaces of an apparent similar smoothness (Figure 4).

Aside from Arnold (1974), which describes and categorizes the tarsal structures of 15 species of cockroach, there is little information on tarsal structure in the cockroach. Arnold (1974) separates the tarsi into three types: type one, where the euplantula and arolium are absent or barely visible; type two, where the euplantula are prominent but the arolium is a small pad between the claws; and type three, where both euplantula and arolium are prominent. Usually cockroaches with type one or two tarsi lack the ability to climb upon smooth surfaces. It appears that *G*. *portentosa* shares the characteristics of type three tarsi, since they demonstrated the ability to climb the smooth surfaces of their tank and to adhere to smooth surfaces during mechanical testing. Furthermore, from analysis of the light microscope and scanning electron microscope pictures, it was clear that both the euplantula and arolium were prominent features of the tarsus. Another feature of the arolium that supports the idea that *G*. *portentosa* possesses a type three tarsi were the “finger-like” projections found in a ridge at the edge of the arolium on the rear leg (Figure 3). Arnold (1974) describes sculpting at the leading edge of the arolium, stating that different species of cockroach possessing the ability to adhere to smooth surfaces had a sculpting of the arolium that took the form of smooth ridges, knobs, or closely adjourning rows of papillae. Arnold (1974) suggested that this sculpting aided the cockroach in the adhesion to smooth surfaces by increasing the surface area available for adhesion. These microstructures are noted in the desert locust, and these structures also are attributed to a greater climbing ability (Kendal 1970). More recently these “finger-like” projections have been seen in other cockroaches that posses the ability to adhere to smooth surfaces with ease (Clemente et al. 2008). However, robust mechanistic hypotheses have yet to be tested on the function of these microstructures.

This study demonstrated that foot morphology plays a key role in substrate adhesion in *G. portentosa*. Foot morphology is thought to relate to ecological niche (Roth and Willis 1952; Arnold 1974), and it appears that the tarsal morphology of *G. portentosa* allows it to climb effectively over a wide range of substrates.
